# Individuals living with a liver transplant – a follow-up study exploring mental, emotional and existential issues 10 years after transplantation

**DOI:** 10.1080/17482631.2023.2233279

**Published:** 2023-07-06

**Authors:** Dagfinn Nåden, Ida Torunn Bjørk

**Affiliations:** a Department of Nursing, Faculty of Health, OsloMet – Oslo Metropolitan University, Oslo, Norway; b Department of Public Health Science, Faculty of Medicine, University of Oslo, Blindern, Oslo

**Keywords:** Liver transplantation, 10 years post-transplant, Existential, Humble attitude, Hopelessness, Dwelling-mobility, Indifferent attitude

## Abstract

**Background:**

Going through a liver transplantation is by many recipients considered mentally and emotionally burdensome.

**Aim:**

The aim of this study was to explore individuals’ mental, emotional and existential experiences living with a liver transplant during a period of approximately ten years.

**Methodological design:**

The methodology in this study is based on Gadamer’s hermeneutics. Galvin and Todres’ conceptual framework on well-being was applied in the interpretation process.

**Research methods:**

Both researchers conducted interviews, which took the form of conversations. We made use of Brinkmann and Kvales’ three types of interpretation.

**Ethical issues and approval:**

The study was approved by the Ombudsman for Privacy of the Norwegian Social Data Services and is based on informed consent and confidentiality.

**Results:**

Three themes emerged through interpretation: 1. From great suffering to gratitude and a humble attitude towards life. 2. From living in uncertainty to leading a normal life. 3. From hopelessness and anxiety to an indifferent attitude towards life.

**Conclusion:**

This study showed that the process of receiving a new liver and living with it, had changed most of the participants’ attitudes towards life in a humble way. Some persons struggled with life and experienced depression anxiety, as well as lack of energy.

## Introduction

Liver transplantation has been a worldwide common life-saving treatment for patients with end-stage liver disease for many decades. Although life expectancy after liver transplantation has increased over the years, longtime survival after 10 and 15 years in Europe is approximately 61% and 51% respectively (ELTR, 2021). The present study is a qualitative follow-up study focusing on psychological experiences of nine patients 10 years after they received a liver transplant. Although most patients increase their psychological quality of life compared to their pre-transplant status (Yang et al., 2014), going through a transplantation process and experiencing life with a transplanted liver is by many recipients considered mentally and emotionally burdensome (Dabrowska-Bender et al., 2016). In addition, a shorter life expectancy than the general population may also create additional emotional and existential reactions.

Detailed searches in databases show that publications from the last 5–6 years on patient experiences after liver transplantation mostly concern experiences with paediatric transplantations and living donor issues. To our knowledge, the studies that do examine adult patients’ personal experiences after transplantation are mainly quantitative studies published before 2016 that explore the patients’ quality of life (QOL) with standardized instruments. Two systematic reviews published within the last 15 years summarize results on long-time experiences and perceptions of QOL in survivors of liver transplantation (Tome et al., 2008; Yang et al., 2014). The review by Tome et al. (2008) included both longitudinal studies that compared patients’ pre- and post-transplant scores on a variety of QOL instruments and cross-sectional studies using the generic health assessment instrument Medical Outcomes Short Form-36 that compared liver transplant patients with controls from the general population. Included studies were published between 1984 and 2006, and average time since transplantation was 25 months in the longitudinal studies and 45 months in the cross-sectional studies. In their conclusion, Tome et al. (2008) highlighted a general increase in QOL scores between pre- and post-transplant assessments, but also that liver transplant patients had significant deficiencies in most QOL domains compared to the general population. They concluded that “the perception of improvement of QOL after LT may have been overstated” (Tome et al., 2008), and suggested that attention in future studies should, among many aspects, be focused on psychological health.

Yang et al. (2014) reviewed 23 studies between 2000 and 2013 that reported on long term QOL in patients more than 5 years after liver transplantation. In their narrative review, they found similar to Tome et al. (2008) that post-transplant QOL generally increased compared to pre-transplant QOL. The improvements gained in the different domains of health persisted over time especially in bodily pain, physical function, mental health and functional status. Ongoing morbidity, constant use of immunosuppression and surgical complications resulted in worse QOL compared to the reference population, especially in physical health. Although the reports on mental health were conflicting, emotional functioning remained satisfactory up to 20 years after transplantation in seven of the studies included (Yang et al., 2014).

There are related findings from previous research where existential issues have been reported because of serious illness and suffering. In a qualitative study about life after cancer treatment Ueland et al. (2020) reported existential experiences as longing to be oneself, longing for relief from suffering, and longing for rootedness. In a study of rural mobility and dwelling amongst rural elders by Todres and Galvin (2012) they suggest that mobility involves all the ways we move both physically and experimentally to connect ourselves to motivated lived possibilities. In a study of transition from hospital to home Dragosits et al. (2023) found that there is still a lack of knowledge with regard to a deeper understanding of existential well-being in the transition process. Bright et al. (2020) studied the experiences of people with aphasia after stroke with focus on hope. Referring to Todres and Galvin (2010) and Galvin and Todres (2011) dwelling mobility theory of well-being, they state that both the ideas of dwelling and mobility resonate with the participants’ narratives of hope, but there was also a co-existing “existential homelessness”. In a study by Larsen et al. (2017) of women’s experience of being diagnosed with systematic lupus erythematosus the essence was found to be a standstill in life. The paper elucidates how existential life phenomena are lived during the course of being diagnosed. The authors conclude that existential phenomena have previously been sparsely linked to a phase during chronic illness.

More than a decade ago, Desai et al. (2008) suggested that we needed to know more about the patients’ perceptions of quality of life as information on morbidity and mortality did not fully reflect the benefits of transplantation. Despite the severity of a transplant operation, the limitations in post-transplant life activities, and the life-long treatment needed after transplantation there are still few studies that detail the patients’ personal experiences after many years of living with a liver transplant. The present study aims to amend this gap in the literature by qualitatively exploring liver transplant recipients’ mental, emotional and existential experiences 10 years after transplantation.

### Aim

The aim of this study was to explore individuals’ mental, emotional and existential experiences living with a liver transplant during a period of approximately ten years.

## Methodology

A hermeneutic approach was used in this study where understanding is the ultimate goal. In hermeneutic interpretation, the parts are understood in the light of the whole, and the whole in the light of the parts. The parts and the whole illuminate one another and make each other explicit (Barbosa da Silva & Andersson, 1993; Gadamer, 1977; Nåden & Eriksson, 2004; Nåden & Bjørk, 2011). Acquisition (Bildung) is necessary to gain universal knowledge in the interpretation process. Acquisition denotes the ability to translate the individual situation, the particular to the universal, and as Gadamer states, a sacrifice of particularity for the benefit of universality (Gadamer, 1960/1999; Nåden, 2010). We have strived towards this goal during the process and tried to permit ourselves to assume the modes of slowness and silence in the hermeneutic position (Nåden, 2010), to allow the subject matter to present itself “in all its otherness” (Gadamer, 1960/2004). Being in these modes we hopefully have been better able to distinguish the essential from the non-essential, and thereby experienced the possibility to wander down the hermeneutic spiral to depths that can be discovered (Nåden, 2010). Bergbom (2007) presents similar thoughts and declares that to be a spectator consists of being completely present and outside oneself, and to a certain degree forgetting oneself in advantage of what one is seeing.

### Participants and location

All together 21 individuals were recruited to be interviewed pre-transplant after being placed on the ordinary waiting list. Fifteen of these individuals were also interviewed post-transplant while still in hospital. The results from these qualitative interviews are reported elsewhere (references inserted when hopefully accepted). Nine of these individuals accepted to be interviewed 10 years after transplantation.

Participants were recruited through the university hospital in connection with a written notice related to their 10-year medical examination, in which a letter from the researchers was enclosed requesting participation in an interview. The letter contained information about the study and the participants signed a consent form which was returned to the hospital. The sample was skewed in relation to gender with only two women included. The average age was 54 years, range 38–67, and the major groups of diagnosis pre-transplant were post-hepatitis cirrhosis and sclerosing cholangitis. The researchers were informed of the date for the 10-year medical examination for those who accepted to participate so the researchers could schedule to meet the participants for interviews.

### Data collection 10 years post-transplant

Both researchers conducted interviews, which took the form of conversations with the systematic use of concise and simple questions from a semi-structured interview-guide (Kvale & Brinkmann, 2009). Examples of topics in the interview guide were: How is your life now? How has your life been for the last 10 years? Was life after the transplant as you thought? Do you feel that the liver transplant has changed you significantly as a human being? What has meant the most to you during this time? How do you see the future? The interviews lasted for approximately one hour, and they were taped and transcribed verbatim.

### Interpretation

The interview material was read several times to get an overview and to form the first impression of the content. During this work we made notes in the margin of the interviews. Thereafter we searched for specific themes in the respective interviews. At the same time, we also read and studied the pre-transplant interviews of the nine participants once more as well as the post-transplant interviews carried out before the participants left the hospital. We did this to consider the participants long way towards recovery. Several of them had been sick for years before they were put on the waiting list. As an example, we discovered one of these journeys as an experience of moving from serious illness to thankfulness and a humble attitude towards life.

In the interpretation, we also made use of Kvale and Brinkmann’s (2009) three types of interpretation, self-understanding, critical common-sense understanding and theoretical understanding. Self-understanding is a condensed form of what the subjects themselves understand to be the meanings of their statements. Critical common-sense understanding may include a wider frame of understanding than that of the participants themselves. At these two levels the strategy helped to structure the interpretations (Nåden & Eriksson, 2004). At the level of theoretical understanding, a theoretical framework was used for interpreting the meaning of the statements (Kvale & Brinkmann, 2009). We found this strategy of interpretation appropriate in this study, especially at the theoretical level where the Well-being conceptual framework of Todres and Galvin (2010) and Galvin and Todres (2011) was applied.

Main elements in this conceptual framework are mobility, dwelling and dwelling-mobility. Todres and Galvin (2010) state that the essence of mobility lies in all the ways in which we are called into the existential possibilities of moving forward with time, space, others, mood and our bodies. The feeling of this “moving forward” is one of energized flow. Further, the authors state that the essence of dwelling lays in all the ways that we existentially “come home” to what we have been given. The feeling of this “coming home” is one of acceptance, “rootedness” and peace. Lastly, Todres and Galvin explain that the deepest possibility of existential well-being lies in the unity of dwelling-mobility. Referring to Heidegger (1993) dwelling-mobility describes both the “adventure” of being called into existential possibilities as well as the “being at home with” what has been given (Todres & Galvin, 2010). The authors offer several kinds of well-being experiences in this conceptual framework in which dwelling, and mobility occur with a number of different emphases. These emphases are formed by the following lifeworld constituents elaborated by Heidegger: spatiality, temporality, intersubjectivity, mood and embodiment.

Due to space considerations and applicability for this study we have chosen to apply three of the components (emphases) of the framework: spatiality, temporality and mood. Dwelling and mobility related to spatiality is experienced of being, as a sense of being home or a sense of adventure, respectively. Related to temporality dwelling may be experienced as a sense of being grounded in the present moment and mobility as a sense of flow and forward movement. Finally, in relation to mood, dwelling involves a sense of peace and mobility a sense of excitement and desire (Todres & Galvin, 2010).

The different kinds of well-being in this conceptual framework are not mutually exclusive. Both researchers interpreted the data material and discussed the themes in the process until consensus was achieved.

### Ethical considerations

The study was approved by the Regional Research Ethics Committee, no. S-02021. The stipulated procedures for gathering data were followed. The participants determined the time and place of the interviews. These were performed in the hospital, in researchers’ offices, at a quiet café or in the participant’s home. A contract was signed between one of our affiliated institutions and a company doing transcriptions concerning anonymity and confidentiality of participants.

## Results

The following themes emerged through interpretation:

- From great suffering to gratitude and a humble attitude towards life
- From living in uncertainty to leading a normal life
- From hopelessness and anxiety of pain to an indifferent attitude towards life

### From great suffering to gratitude and a humble attitude towards life

Several of the patients felt gratitude towards life after years of serious disease and great suffering. However, the way forward to such recognition was quite different for them. One of the participants lived with major problems before the liver transplantation. After surgery this person experienced to receive life again and that she had a lot to live for. However, after a while she went into a deep depression that she did not understand since she really was so happy to get her life back. In another interview, she looked back in time thinking of the great problems she had in connection with her reaction to symptoms of rejection of the new liver. She said: *Then I felt like I received life again even once more … I have so much to live for, and now I feel I’ve moved back to life*. She said that she has behaved well: *I felt it worked out very well until it was too much because at that time, I thought I was the same as before. So, I started renovating the house, painting and doing a lot of other things. So, I went into a deep depression*. She did not understand her own reaction: *because I was so lucky to have had my life back … I needed to change my mindset … I just lay at home and cried; it was terrible.*

After ten years this woman still has a humble attitude to life: *I feel very well now … And I have for a long time … I was very keen to exercise again. Because when you get such things (liver transplant), you feel like you’re receiving life again. Because I was not exactly very well before surgery … Of course, you do everything which is possible to recover and be as healthy as possible … I feel that I do not live if I don’t go for a walk every day.*

In addition to a humble attitude, she also looked at life in a different way: *At the same time, I have learned to appreciate life in a completely different way. Trifles are nothing to care about … People complain about nothing … Now it is the present that matters. To make every day a celebration. So, I feel that I have learned a lot from it.*

Looking back on the ten years since transplantation, she said: *I never dreamed about it. That it would be so good. Had never imagined that it should turn out that well. So that’s amazing*. Participant 2.

Another participant had waited for liver transplantation for seven years. From the day he was called to go to the hospital for surgery, he felt relief to finally get started. A long time before admission, he gradually became worse. *When I was admitted on Saturday night and got ready, I had no fear of surgery at all. I was just relieved. I was completely calm*. Except for the fact that he had to undergo a minor surgery after the transplant, the postoperative course went well for him. To overcome problems, the strength lies in the ability to laugh, according to the participant.

In an earlier interview after the liver transplant, he said that for him everything really has been a boon. He said he has really put this behind himself: *So, I’ve basically forgotten it … As I say, I’ve distanced myself quite a bit from my entire stay. For now, I feel I’m done with it. Now I’m thinking why I’m at all here* (at the hospital for control and medical examination). At the end of the interview, the participant expressed a positive attitude towards life: *Now it is only one way forward. Now it is only good times. Now it is just to move on, as far as it lasts. It will last for a long time now!*

Ten years after the liver transplant, the participant claimed that he did not have any physical retardations. Still, he said it was quite bad psychologically the first few months. In particular, he mentioned the period before he started working again: *Then I knew that when I lay in the evening, and the time reached three or four o’clock in the morning, when I woke up and everything was bad. And it was! … . Then I was worried about many strange things.*

However, the participant thought about the time before surgery when he himself was ill and did not see how sick the other patients at the hospital ward were. It was an awakening for him seeing the others, and he said: *When I am at medical check-up at the hospital, and look at the grey faces of those who are sick … I think: Oh, did I look like that? I didn’t realize that before. When I was sick and hospitalized myself, I didn’t see in the same way how sick the others were. I think my focus was more on myself. When I sit there and look, I think: Why do they set aside two entire days for me? It’s crazy. We could have done this in an hour, but I’m sitting here for two days. Look at these poor wretches, can’t you help them instead?* Participant 4.

One of the participants was denied liver transplantation in another country. She had to change her thoughts about dying to a new possibility of liver transplantation. But it was hard to change: *The thoughts never gave me peace* … *Thoughts shattered in my head all the time*. After receiving new information of the possibility of a transplantation, she thought: *What can go wrong and what can go right, infection and whatever it may be. I tried to be optimistic, but it was not always easy … it was overwhelming*. But the liver transplant went well: *That’s what I am concerned about, that everything works. It turned out very well … I say that I could not have been better …* After ten years she is thankful and lucky: *I try to be positive … To get up in the morning with a good attitude … Giving up is not possible, then I will be even worse … So, I think you must go on … I have had a very good life … Concerning the liver, it has been stable nearly almost all the time … I have been very lucky! And I mean it … I received the liver transplant and survived so well. I experience that I have had very good health afterwards! … I think the whole process has changed me*. Participant 1.

Another participant was surprised how reduced one became after liver transplantation. He thought it would take years to recover. But that did not happen. *I had no ailments, everything went well*. After the ten-year medical check-up, he said:

*It was perfect in every possible way. Everything was great really*. Concerning the future, he said: *I’m 56 years old. I have had a good life, even though I do not have so many diplomas … So, if it should happen again (new liver), I do not see it as any crisis. Then I’m over 60 years old and have had an exciting, maybe different life. I have to say that I’m really satisfied*. Participant 3.

One of the participants said after being put on the waiting list that he often thought that it might go wrong. After transplantation he had some demanding days when the liver tests rose and when the Solumedrol cure did not work. It was a mental blow. After some time of recovery, he also felt a little more emotional: *When I looked at the children on our national day, I got a lump in my throat, I remember. Thoughts of not living so long had not earlier been conscious. I think that I have a full life.*

Meeting him 10 years after the transplant, he exhibits a humble attitude to life and to the donor: *I do not like wine, but I like whisky. So, I take a small whisky a seldom time. I always take it together with food. If anyone mentions this, I usually say that drinking a large amount of alcohol would be like spitting on the grave to him that I got the liver from … I think so much about the fact that someone else had to die for me to live*. He continues: *In the movie “Saving Private Ryan” the entire squad dies to save the guy Ryan. Then at the end of the movie, the officer is deadly wounded, so he says to Ryan: “*Earn it!” *I think about the whole way of life … Occasionally I ride up to the mountains. There is a very nice view. So, I usually stop there, leaning against the railings and watching the view. And then I tend to say … and I’m not religious … but when I ride from there I say, Thank you. “Thank you!” [to his donor]*. Participant 7.

After 10 years since liver transplantation the participants lived a good life. They were thankful and had a humble and positive attitude towards life, feeling they had received life again. Another common trait was that the process of receiving a new liver and the subsequent recovery process changed them in different ways during their adventurous journeys.

### From living in uncertainty to leading a normal life

Living with a serious liver disease is living in uncertainty. One of the participants said that you never know when the problems are coming: *I feel I’ve had a good time. However, I had over sixty days in hospital in 1999, and then you get a little downturn psychologically of course. One day I called my employer at half past six in the evening and said I will be back tomorrow at eight o’clock. But then I was admitted to the hospital that evening.*

The participant was not prepared for a transplant. He said that it is too bad to have a new liver because he is not an old guy. He continues: *But on the other hand, I see that I have no choice. And of course, it’s a little shock when you hear the message that you must get through this. But one just has to try to get through it … . They found cellular changes in the gall bladder, which is a kind of cancer. And then the choice was very easy: There is no choice!*

When being on the way to the operating room he said to the nurses: *“I don’t like it, but there is no choice”*. Later: *I woke up and thought: Now it is done! … I had a rejection of the liver, and that was a huge downturn.*

Ten years after the liver transplant the participant says he tries to live as normally as possible: *There is nothing more to say than that everything works as it should … I’m in a full job … It has really gone very well … I have bicycled a few times. … But if I ride too hard, I drive my body and get terribly tired … I’m able to push my body, but then it takes a lot longer to recover.*

He does not think that the liver transplant has changed him so much, and claims that when so many years pass, you will go back to normal again: *One slides over to the normal, really*. He has not had many thoughts about the future: … *if everything works as it should … have not thought so much about this … But it is clear … somebody must come back here after a certain period and have a new liver transplant … we have been told this is something we should not think about, and I do not. But, of course, it’s “lying” there. You have someone else’s organ in your body*. Participant 5.

Another participant suffered from a serious liver disease for a short period before liver-transplantation and was anxious about the liver transplant not turning out well. He states that *it doesn’t matter whether ninety percent of transplantations turn out well if I’m one of the ten percent where it does not work well … People say that one must be optimistic, but at the same time I’m a realist and know what can happen. … My nature is not just being born an optimist.*

After a successful liver transplantation, the participant experienced two rejections while admitted to hospital: *Then I became really depressed*. Another new experience for the participant was the handling of emotions: *Not managing my own feelings is new for me. I can start crying for nothing*. This still applies ten years after the operation. He is also very restless. Apart from a minor depression a few years ago, he has lived an active and normal life in full employment. Participant 6.

Even though these two participants experienced uncertainty before and after liver transplantation, they live an active and normal life.

### From hopelessness and anxiety of pain to a more or less indifferent attitude towards life

Short time after transplantation one participant had to undergo new surgery: *I was a little sad to hear that I had to undergo new surgery … One becomes a little depressed.*

The first six-seven years after the transplantation of a new liver were rather good for the participant. But he never reached the same energy level as before transplantation: *I didn’t have the energy to start in a job again*. He has been in a bad situation the last three years. The liver has not functioned as it should because the liver “seals” the biliary ducts: *I have now the same illness as I had ten years ago … I lose strength*. He is therefore under examination for a new liver transplantation. Because of pain when eating and exercising, it has been a difficult life: *What I want to do, I cannot manage to do any more*. *… I haven’t been working full time the last ten years. My energy is lost! … If I do a thing today, I wouldn’t manage anything tomorrow. It is not possible to sleep off this fatigue*. The participant states that there might be a possibility that there is cancer in the liver: *Then you experience a shock. How the situation is now, life stands still, one only exists*. It also becomes difficult for the patient when the health personnel do not meet the person in need in a dignified way.

He has been through a journey for the past ten years, from anxiety to transplant and subsequent pain, being sad and vulnerable to the message of new surgery because of cancer in the bile duct. His mood fluctuates in line with good or bad news. But he is also of the opinion that it is worthwhile to spend months in hospital when life can be saved. Occasionally he becomes restless. The disease has caused him to be in shock nearly all the time.

The situation in which the participant has been in recent years has led to a more indifferent attitude towards life: *I don’t think that the disease itself has changed me, but I have become more indifferent to what happens*. Participant 8.

One of the participants had problems with the liver half a year after transplantation. A feeling of lethargy began three months after surgery. He has also developed a headache due to a blow to the head that resulted in injuries two and a half years ago. The participant claims that the blow to the head was based on racism. Ten years after surgery he says: *Life has changed, but not because of the liver transplant. Now I walk 50 metres and then sit down … I cannot go the mountains. I remember old days when I was in the mountains. Now I cannot go … Worst is the lack of energy*. He also says: *My wife has been affected by cancer; she cries a lot at home. We are depressed*. Participant 9.

Hopelessness and anxiety because of loss of energy and a life that stands still has led to a more or less indifferent attitude to life.

## Theoretical interpretation and discussion

Most of the participants felt gratitude ten years after the liver transplant, and even during most of the time since surgery. Many of the participants had difficulties before and after liver-transplantation, including depression. As time went by, their views on life changed.

They have gained a more overall perspective of both their own lives and life in general, and they appreciate life in a completely different way.

The participants have been given the gift of life, feeling humility and gratitude. The results of this present study are largely in line with Wright et al (2017, 2018). operationalization of the core of humility as low self-focus and high other-focus. These authors state that being “epistemically aligned” results in a reduced sense of “ego” – a reduced attachment to one’s “self” and its products and capacities. This simply means that the person holds his beliefs, values, skills etc. in perspective and what matters is the things that can be accrued by and accomplished with them. An ethically aligned attitude, meaning that humility is the experience of “all else” – e.g., a vast web of interconnected beings whose needs are as morally relevant, as worthy of attention and concern as one’s own (Wright et al., 2018) becomes visible as well. A shift in perspective is also at the forefront when participants look at themselves in the light of other suffering human beings. Wright et al. (2017) found humility to be positively related to a wide range of morally relevant attributes or qualities, such as civic responsibility, gratitude, humanitarian-egalitarian attitudes, empathy, moral identity, integrity, benevolence and “moral foundation” intuitions. Dimensions of generosity to be related to humility is demonstrated by Exline and Hill (2012). These qualities are particularly evident in theme one.

Participants expressed both humility and gratitude. Kruse et al. (2014) found that humility and gratitude are mutually reinforcing. Likewise, expressing a state of gratitude increases emotional well-being and life satisfaction. Those participants in Kruse et al.’s study who wrote a letter expressing their gratitude showed higher humility than those who wrote about a neutral activity, and people’s baseline humility predicted the degree of gratitude felt after writing the letter. Our results are also in line with Wood et al. (2010) study, that gratitude may include a general appreciation for life. Expressing a state of gratitude increases emotional well-being and life satisfaction (Emmons & McCulluogh, 2003). The results of this present study have great similarities with Wayman and Gaydos (2005) results in the study of self-transcending through suffering, where gratitude and humility are key attitudes. We found, like Wayman & Gaydos, that they valued their lives much more than they did before their suffering. Most of the participants in this present study state 10 years after transplantation that they really have had a good life in many ways. The participants have experienced much suffering, some of them for years before and after the liver transplant. Most of the participants experienced suffering on the ontological level (Eriksson, 2006, 2018), where the human being is in deepest contact with him- or herself. Some have travelled a long way to gain reconciliation and renewed health.

In their theory Todres and Galvin (2010) and K. T. Galvin and Todres (2011) offer various levels and kinds of well-being related to dwelling and mobility. Especially in theme one, we acknowledge that dwelling is experienced in a spatial way, where one feels a sense of “being at home”, described by a participant as appreciating life in a completely different way. According to Galvin and Todres (2011) well-being as a sense of at-homeness is anything that offers a place of settling or peace. Furthermore, Todres and Galvin (2010) state that dwelling emphasizes a settling into the present moment with its acceptance of things as they are. Bright et al. (2020) suggest that holding elements of the past, present and future may help people to have a sense of “dwelling”. They refer to Todres and Galvin (2010) who underline that this is “a form of being grounded in the present moment, supported by a past that is arising, and openness of a future that is calling”.

Several of the testimonies referred to in the results are interpreted as spatial dwelling of at-homeness. Likewise, we acknowledge that when mobility is experienced in a spatial way, one has a sense of adventure, expressed in simple words by one participant, as making every day a celebration. Especially important is the spatial mobility of adventurous horizons, which might simply be a sense of horizon in which a person experiences the feeling of mobility by looking out at the stars or a sunset. An example of this mobility is the participant who stopped the bike for a rest in the mountains and looked at the beautiful view being thankful for his good life. We interpret this as in line with Todres and Galvin (2012) who state that an understanding of the meaning of mobility might be extended to include an aesthetic dimension. A sense of adventurous horizon is anything that offers a place of promise (Galvin & Todres, 2011).

There is also a spatial dwelling-mobility that Galvin and Todres (2011) call abiding expanse, where the person is tuned into the spatial possibilities of their environments that combines “settled at-homeness” as well as “adventurous horizons” (Todres & Galvin, 2010), either metaphorically or literally in the ways that they are valued and wanted (Galvin & Todres, 2011). Well-being as a sense of abiding expanse is any place that stretches between home and adventure (Galvin & Todres, 2011), which is made visible in the results through several of the participants’ stories, as in the person who says that she does not feel that she is alive if she does not go for a walk every day. Also, we can read the adventurous horizons and at-homeness, at least metaphorically, in one participant’s words that it is the present that matters.

When experiencing dwelling in a temporal way, there is a sense of being grounded in the present moment (Todres & Galvin, 2010), a well-being experience that emphasizes present-centredness (Galvin and Todres (2011). It might be an intimacy, a sense of belonging or a deep connection with what is happening in the moment (Galvin & Todres, 2011). We interpret results of the present study, especially the first two themes of the study, to be related to or fall within the present-centredness of dwelling. Participants are “brought home” to the very simple event of “just being”. There is a completeness and satisfaction in this moment of temporal dwelling, as Galvin and Todres (2011) state. This temporal dwelling might be illuminated by one of the participants’ statements that when he looked at the children on our national day, he got a lump in his throat, or it might be as simple as getting up in the morning with a good attitude, as one participant expresses.

When mobility is experienced in a temporal way, there is a sense of temporal “flow” and forward movement (Todres & Galvin, 2010), illustrated by participants in words like giving up is not possible, that will be even worse, or if needing a new liver once more, it is no crisis. Concerning temporal mobility, the authors also states that a person may be energized by the possibilities that call from the future and that motivate his and her in a way that constitutes a sense of purpose (Galvin & Todres, 2011). This is evident in many of the participants of this study, especially in those who represent theme one and two of the results. It might, as Galvin and Todres (2011) state, represent a flow and continuity to the ongoing progression of one’s life in time, in our case from great suffering to gratitude and a humble attitude towards life. It may be illustrated by one participant empathizing that it is only one way forward and only good times, it is just to move on.

This theory of well-being also points at a temporal dwelling-mobility called renewal, that indicates something of the freshness, aliveness and uniqueness of the present moment which has never before quite happened like this, as well as a sense of possibility and potential movement that leans towards a future that opens up (Galvin & Todres, 2011). This thought applies, in different ways, to many of the participants in this study, such as having a good life, while at the same time they must go on. The authors state that well-being as a sense of renewal is any welcome “joining” between the depth of the present and the openness of the future. This kind of well-being is best visualized in theme one and two.

Results might also be seen in the light of dwelling experienced as mood. Galvin and Todres (2011) state that there may be a sense of peacefulness when one has fulfilled a task or responsibility that required some effort and commitment. Effort and commitment largely apply to people who have undergone a demanding time before the liver transplant as well as the recovery afterwards, and then live a good life for years. Dwelling as a sense of peacefulness and thankfulness is illustrated by one participant thinking of the fact that the donor had to die for him to live.

When mobility is experienced as mood there is a sense of excitement or desire (Todres & Galvin, 2010). A sense of excitement or desire in a metaphorical sense, may, according to Galvin and Todres (2011) occur when there is a feeling of possibility, when the world is inviting one into horizons that connect with the desires of one’s heart. This mood might be illuminated by one of the participants thinking about the whole way of life, expressing gratitude to his donor. Galvin and Todres talk about this mood as a kind of “life force” or vitality. The distinction between mood mobility and mood dwelling might seem difficult to distinguish, and in our case these types of mood might be seen as not mutually exclusive.

Also concerning mood there is a well-being that can hold both mood mobility and mood dwelling together. This is an experience that can straddle both excitement or desire and peacefulness. Galvin and Todres (2011) call this mood dwelling-mobility a mirror-like multi-dimensional fullness.

This is a well-being experience characterized by a highly complex mood that participates in both the energetic quality of enthusiasm and interest as well as the settled quality of being at home with oneself and the world (Galvin & Todres, 2011). This is a fullness of mood that can be many things, such as sadness, love and happiness because this mirror-like mood does not need to separate itself from whatever is happening right now. We acknowledge these different dimensions in one participant being happy to receive life again for thereafter to go into a deep depression. We can to a certain extent sense this dwelling-mobility of mirror-like multi-dimensional fullness also in other participants’ stories when they talk about their lives ten years after liver transplantation.

The third theme contrasts with themes one and two in that it deals with suffering, lack of energy, anxiety, sadness and hopelessness related to the new liver or personal matters. Losing strength makes it impossible to work, and what the participants want to do, they cannot do because of a lack of strength. Doing things one day makes it impossible to do things the next day. Sleeping off the situation is impossible. A feeling arises that life stands still, that one simply exists. Hopelessness about one’s own situation leads to indifference. This result is compatible with Larsen et al. (2017) study on women’s experience of being diagnosed with systemic lupus erythematosus, where the main theme was just being at a standstill in life. In a study on longing Ueland et al. (2020) argue that because one does not feel at home in one’s life, there may not be any firm rooting from which to start one’s longing. It may also be difficult to see clearly what life is like and what one longs for because one feels different and there are significant health challenges to handle in everyday life. The results from these two studies are compatible with Galvin’s text where the author gives examples of issues in the everyday realm of care delivery, as having to leave life, never to be the same; and care contexts where death is sometimes close or always present as in intensive and critical care (Galvin, 2021). Dragosits et al. (2023) report findings from a study of older patients and their relatives’ descriptions of suffering and well-being in the transition from hospital to home. They found that time was experienced with anxiety about handling future care and daily demands. The authors refer to Galvin and Todres (2013) who place this kind of suffering in the existential dimension temporality. This is in line with the third theme in this present study, where patients stand still, and the mood fluctuates in line with good and bad news. Dragosits et al. further state that fear of the unknown can be seen as fertile soil for existential suffering.

Eriksson (2006, 2018) asserts that in the deepest sense suffering is a kind of “dying”. The author refers to Pauli (1938) who describes that suffering can be like a temptation, which can easily create bitterness and indifference for everything and everyone. Nuances of this indifference can be seen in the third theme. Concerning this aspect Eriksson (2006, 2018) points out that a suffering human being must receive confirmation of his or her dignity as a human being. Confirming the person’s absolute dignity is to help the person to experience hope and future possibilities. Bergbom et al. (2021) state that a caring relationship may facilitate the movement and development towards “the person one really wants to be” if carers can give time and space for conversations or just “being there”, and this can give serenity to those who are suffering.

In line with these thoughts, Galvin and Todres (2011) maintain that when there is a sense of future orientation, a person may be tuned into the temporal possibilities of moving forwards into the future. They further state that without this possibility one may feel “stuck”, as if frozen in time without meaningful invitations “into the future”. The participants representing theme three seem deprived of the opportunity to move forward in life because of their health condition or because of not having received sufficient or adequate support in their life and therefore cannot see future possibilities. Eriksson (2006) emphasizes that in such an alleviation and reconciliation process where suffering is given meaning, suffering can transform into endurable suffering.

## Strengths and limitations

The strength of this study is that both researchers have been involved in both interviewing the participants and interpretation of the data. The participants were interviewed before, after and lastly ten years after transplantation by the same researchers at all times. This procedure was chosen because we could then use our previous knowledge of data from the respective participant and thus build on established contact. A limitation of this study may be the skewed sample with only two women among the recruited persons.

## Conclusion

This study shows that the process of receiving a new liver and living with it has changed the participants’ attitudes towards life in a humble way. They claim that they are living good lives. Even though most of the participants experience living good lives 10 years after the liver transplantation, there are some who struggle with life. There is therefore a need for further research in the areas of those persons who have feelings of lethargy, lack of energy and depressions, with the aim of gaining knowledge about how these people can best be helped when life seems hopeless and when indifferent attitudes towards life occur.

## Implications for practice

This study shows that most people who undergo a liver transplant have lived good lives related to mental, emotional and existential issues the first ten years after the transplant. The study also shows that some persons have problems and difficulties in life after the transplant. There is therefore a need to heighten awareness among healthcare personnel about problems in the years to come concerning the mentioned issues. Galvin (2021) points to existential resources that are up to the task of guiding the head, the hand and the heart of care, thereby addressing human existence. The author underscores that a foundational point is that there is always some freedom, and always some possibility for moving forward as well as for settling in any condition. Health personnel at hospitals and healthcare centres who meet people in the years after transplantation have a special responsibility in caring for these patients in appropriate ways to ensure that they receive the best possible treatment and follow up in relation to issues in the years after the transplant.

## References

[cit0001] Barbosa da Silva, A., & Andersson, M. (1993). *Vetenskap och människosyn i sjukvården: En introduction til vetenskapsfilosofi och vårdetik [The role of science and the view of man in professional caring: An introduction to philosophy of sciences and healthcare ethics]*. Svenska hälso- och sjukvårdens tjänstemannaförbund.

[cit0002] Bergbom, I. (2007). Klinisk vårdvetenskap – hermeneutiska observationer (Clinical caring science – hermeneutic obserservations). In K. Eriksson, U. Å. Lindström, D. Matilainen, & L. Lindholm (Eds.), *Gryning III. Vårdvetenskap och Hermeneutik [Dawn III. Caring science and Hermeneutics]* (pp. 61–11). Department of Caring Science, Åbo Akademi University.

[cit0003] Bergbom, I. L., Lindström, U. Å., Eriksson, K., & Nåden, D. (2021). Understanding the meaning of doubt, despair, and belief in caring sciences. *International Journal for Human Caring*, 25(2), 89–99. 10.20467/HumanCaring-D-20-00031

[cit0004] Bright, F. A. S., McCann, C. M., & Kayes, N. M. (2020). Recalibrating hope: A longitudinal study of the experiences of people with aphasia after stroke. *Scandinavian Journal of Caring Sciences*, 34(2), 428–435. 10.1111/scs.12745 31487069 PMC7432176

[cit0005] Dabrowska-Bender, M., Michalowitcz, B., & Paczek, L. (2016). Assessment of the quality of life in patients after liver transplantation as an important part of treatment results. *Transplantation Proceedings*, 48: 1697–1702. 10.1016/j.transproceed.2015.12.139 27496474

[cit0006] Desai, R., Jamieson, N. V., Gimson, A. E., Watson, C. J., Gibbs, P., Bradley, A., & Praseedom, R. K. (2008). Quality of life up to 30 years following liver transplantation. *Liver Transplantation*, 14(10), 1473–1479. 10.1002/lt.21561 18825684

[cit0007] Dragosits, A., Martinsen, B., Hemingway, A., & Norlyk, A. (2023). Being well? A meta-ethnography of older patients and their relatives’ descriptions of suffering and well-being in the transition from hospital to home. *BMC Health Services Research*, 23(1). 10.1186/s12913-023-09039-w PMC9901096 36747154

[cit0008] ELTR. (2021). eltr.org,Retrieved March , 2021.

[cit0009] Emmons, R. A., & McCulluogh, M. E. (2003). Counting blessings versus burdens: An experimental investigation of gratitude and subjective well-being in daily life. *Journal of Personality and Social Psychology*, 84(2), 377–389. 10.1037/0022-3514.84.2.377 12585811

[cit0010] Eriksson, K. (2006). *The suffering human being*. Nordic Studies Press.

[cit0011] Eriksson, K. (2018). *Vårdvetenskap. Vetenskap om vårdandet. Om det tidlösa i tiden. [Caring Science. The Science of caring. About the timeless in time]*. Liber.

[cit0012] Exline, J. J., & Hill, P. C. (2012). Humility: A consistent and robust predictor of generosity. *The Journal of Positive Psychology*, 7(3), 208–218. 10.1080/17439760.2012.671348

[cit0013] Gadamer, H.-G. (1960/1999). *Truth and method*. (Joel Weinsheimer and Donald G. Marshall, Translation by). Continuum.

[cit0014] Gadamer, H.-G. (1960/2004). Sandhed og metode. Grundtræk av en filosofisk hermeneutik [Truth and method. Basic features of a philosophical hermeneutics]. In *Translation, introduction and notes by Arne Jørgensen*. Systeme Academic.

[cit0015] Gadamer, H.-G. (1977). Vom zirkel des verstehens [The circle of understanding. In H. G. Gadamer (Ed.), *Kleine Schriften* (Vol. 4, pp. 54–61). Mohr.

[cit0016] Galvin, K. (2021). ‘Getting back to the matters’: Why the existential matters in care. *Scandinavian Journal of Caring Sciences*, 35(3), 679–684. 10.1111/scs.13022 34390265

[cit0017] Galvin, K., & Todres, L. (2011). Kinds of well-being: A conceptual framework that provides direction for caring. *International Journal of Qualitative Studies on Health and Well-Being*, 6(4), 10362. Epub 2011 Dec. 9. 10.3402/qhw.v6i4.10362 PMC3235360 22171222

[cit0018] Galvin, K., & Todres, L. (2013). *Caring and well-being: A lifeworld approach*. Routledge.

[cit0019] Heidegger, M. (1993). Building, dwelling, thinking. In D. F. Krell (Ed.), *Basic writings: Martin Heidegger* (pp. 343–364). Routledge.

[cit0020] Kruse, E., Chancellor, J., Ruberton, P. M., & Lyubomirsky, S. (2014). An upward spiral between gratitude and humility. *Social Psychological and Personality Science*, 5(7), 805–814. 10.1177/1948550614534700

[cit0021] Kvale, S., & Brinkmann, S. (2009). *Interviews. Learning the craft of qualitative research interviewing*. Sage Publications, Inc.

[cit0022] Larsen, J. L., Hall, E. O. C., Jacobsen, S., & Birkelund, R. (2017). Being in a standstill-of-life: Women’s experience of being diagnosed with systemic lupus erythematosus: A hermeneutic-phenomenological study. *Scandinavian Journal of Caring Sciences*, 32(2), 654–662. 10.1111/scs.12491 28699208

[cit0023] Nåden, D. (2010). Hermeneutics and observation – a discussion. *Nursing Inquiry*, 17(1), 75–81. 10.1111/j.1440-1800.2009.00472.x 20137033

[cit0024] Nåden, D., & Eriksson, K. (2004). Understanding the importance of values and moral attitudes in nursing care in preserving human dignity. *Nursing Science Quarterly*, 17(1), 86–91. 10.1177/0894318403260652 14752958

[cit0025] Nåden, D., & Bjørk, I. T. (2011). Patients' experiences in hospital following a liver transplantation. *Scandinavian journal of caring sciences*, 26(11), 169–177. 10.1111/j.1471-6712.2011.00911.y 21812799

[cit0026] Pauli, E. (1938). *Kamp eller resignation [Struggle or resignation]*. Hagerup.

[cit0027] Todres, L., & Galvin, K. (2010). “Dwelling-mobility”: An existential theory of well-being. *International Journal of Qualitative Studies on Health and Well-Being*, 5(3), 5444. 10.3402/qhw.v5i3.5444 PMC2938924 20842215

[cit0028] Todres, L., & Galvin, K. (2012). “In the middle of everywhere:” a phenomenological study of mobility and dwelling amongst rural elders. *Phenomenology & Practice*, 6(1), 55–68. 10.29173/pandpr19854

[cit0029] Tome, S., Wells, J. T., Said, A., & Lucey, M. R. (2008). Quality of life after liver transplantation. A systematic review. *Journal of Hepatology*, 48(4), 567–577. 10.1016/j.jhep.2007.12.013 18279999

[cit0030] Ueland, V., Rørtveit, K., Dysvik, E., & Furnes, B. (2020). Life after cancer treatment – existential experiences of longing. *International Journal of Qualitative Studies on Health and Well-Being*, 15(1), 1838041. 10.1080/17482631.2020.1838041 33112718 PMC7599008

[cit0031] Wayman, L. M., & Gaydos, H. L. B. (2005). Self-transcending through suffering. *Journal of Hospice and Palliative Nursing*, 7(5), 263–270. 10.1097/00129191-200509000-00013

[cit0032] Wood, A. M., Froh, J. J., & Geraghty, A. W. (2010). Gratitude and well-being: A review and theoretical integration. *Clinical Psychology Review*, 30(7), 890–905. 10.1016/j.cpr.2010.03.005 20451313

[cit0033] Wright, J. C., Nadelhoffer, T., Perini, T., Langville, A., Echols, M., & Venezia, K. (2017). The psychological significance of humility. *The Journal of Positive Psychology*, 12(1), 3–12. 10.1080/17439760.2016.1167940

[cit0034] Wright, J. C., Nadelhoffer, T., Ross, L. T., & Sinnott-Armstrong, W. (2018). Be it ever so humble: Proposing a dual-dimension account and measurement of humility. *Self and Identity*, 17(1), 92–125. 10.1080/15298868.2017.1327454

[cit0035] Yang, L. S., Shan, L. L., Saxena, A., & Morris, D. L. 2014. Liver transplantation: A systematic review of long-term quality of life. *Liver International*, 34(9), 1298–1313. 10.1111/liv.12553 24703371

